# Effect of Antibiotics on the Colonization of Live Attenuated *Salmonella* Enteritidis Vaccine in Chickens

**DOI:** 10.3389/fvets.2021.784160

**Published:** 2021-12-01

**Authors:** Jiangang Hu, Chuanyan Che, Jiakun Zuo, Xiangpeng Niu, Zhihao Wang, Liyan Lian, Yuanzheng Jia, Haiyang Zhang, Tao Zhang, Fangheng Yu, Saqib Nawaz, Xiangan Han

**Affiliations:** ^1^Shanghai Veterinary Research Institute, The Chinese Academy of Agricultural Sciences, Shanghai, China; ^2^College of Animal Science, Anhui Science and Technology University, Chuzhou, China

**Keywords:** *Salmonella* Enteritidis, live vaccine, antibiotics, colonization, bacterial isolation

## Abstract

Salmonellosis, caused by *Salmonella* Enteritidis, is a prevalent zoonosis that has serious consequences for human health and the development of the poultry sector. The *Salmonella* Enteritis live vaccine (Sm24/Rif12/Ssq strain) is used to prevent *Salmonella* Enteritidis around the world. However, in some parts of the world, poultry flocks are frequently raised under intensive conditions, with significant amounts of antimicrobials to prevent and treat disease and to promote growth. To investigate whether antibiotic use influences the colonization of orally administered *Salmonella* live vaccines, 240 1-day-old specific pathogen-free chicks were randomly divided into 24 groups of 10 animals for this study. The different groups were treated with different antibiotics, which included ceftiofur, amoxicillin, enrofloxacin, and lincomycin–spectinomycin. Each group was immunized 2, 3, 4, and 5 days after withdrawal, respectively. At 5 days after immunization, the blood, liver, and ceca with contents were collected for the isolation of the *Salmonella* live vaccine strain. The result showed that no *Salmonella* vaccine strain was isolated in the blood and liver of the chicks in those groups. The highest number of *Salmonella* vaccine strains was isolated in the cecum from chicks vaccinated 2 days after ceftiofur withdrawal, and no *Salmonella* vaccine strain was isolated from the cecum in chicks immunized 3 days after ceftiofur withdrawal. Among the chickens immunized 4 days after the withdrawal of amoxicillin, enrofloxacin, and lincomycin–spectinomycin, the number of *Salmonella* vaccine colonization in the cecum was the highest, which was higher than that of the chickens immunized at other withdrawal interval (2, 3, and 5 days) groups and was higher than that of the chickens without treatment (*P* < 0.05). This study provides a reference for the effective use of the *Salmonella* Enteritidis live vaccine and key antibiotics commonly utilized in the poultry industry.

## Introduction

Salmonellosis is a serious public health and veterinary problem (1). Since the 1980s, *Salmonella* Enteritidis (*S*. Enteritidis) causes most of food-borne human *Salmonella* outbreaks. The organism can be isolated from a variety of foods, including poultry meat, eggs, pork, and dairy products (2). Annually, an estimated one million cases of human salmonellosis occur in the USA. This was the most common infection reported, with the highest number of associated hospitalizations and deaths (17.6 illnesses per 100,000 persons), and *S*. Enteritidis was the most common serotype (22 percent) (3). Furthermore, as a zoonosis, the reservoir of the bacteria consists primarily of domesticated fowl and their products (4–7).

When transmitted horizontally, *Salmonella* enters the body through the fecal–oral route, invades the intestinal tract, reaches the liver and spleen in a matter of hours, and can colonize the ceca in chickens. *Salmonella* is an intracellular bacterium which cannot be tackled with antibiotics, and once birds are infected, they become life-long carriers. *Salmonella* Enteritidis is the most prevalent serovar worldwide, compromising the reputation of the poultry industry and commonly associated with food-borne outbreaks endangering human health. Vaccination, on the other hand, is one of the most promising control strategies for reducing *Salmonella* in chickens (8). Among the vaccines against *Salmonella* Enteritidis, live vaccines have better immune effects than inactivated vaccines. Previous research with this Sm24/Rif12/Ssq vaccine strain demonstrated a significant reduction in internal *S*. Enteritidis egg contamination after vaccination (3). This vaccine strain can be administered orally to immunize newly hatched chickens, and it colonizes the gut extensively and prevents re-infection by other *Salmonella* strains by a genus-specific mechanism (3). However, immunization of young chicks during the first week alone is not effective enough, as their immune system has not fully developed (9). Furthermore, in different parts of the world, poultry flocks are frequently raised under intensive conditions, with large amounts of antimicrobials used to prevent and treat disease as well as to promote growth. Medication for broiler chicks under 3 days of age is common in the Middle East and East Asian countries to eliminate vertical or hatchery-transmitted bacterial pathogens (10). The issue of ceftiofur resistance was raised more than a decade ago, and some hatcheries had already voluntarily stopped using ceftiofur in favor of lincomycin–spectinomycin (11). The impact of discontinuing ceftiofur and replacing it with the antibacterial combination of lincomycin–spectinomycin, which is a regular practice in the industry, is still unknown. Amoxicillin is an antibiotic with a broad spectrum of action that belongs to the class of penicillins and the subclass of aminopenicillins (12). Enrofloxacin, a quinolone developed specifically for animal usage, exhibits broad antibacterial activity. It is a potent antibiotic with wide antibacterial action against gram-positive and gram-negative microorganisms (13). The antibiotics ceftiofur, amoxicillin, enrofloxacin, and lincomycin–spectinomycin are typically used to treat chick infections with poultry pathogens, including avian pathogenic *Escherichia coli, Salmonella, Pasteurella multocida, Avibacterium paragallinarum, Bordetella avium, Clostridium perfringens*, and *Riemerella anatipestifer, etc*. (14). Hence, newly hatched chickens will be treated with antibiotics to prevent a pathogen infection. However, there is no research on whether the above-mentioned antibiotic treatment affects the colonization effect of the *Salmonella* live vaccine.

In this context, it is critical to understand the effects of antibiotics on the colonization of live *Salmonella* vaccine. In the current study, chickens were administered with an aqueous antibiotic solution by drinking or intramuscular injection to disrupt the ecological balance of intestinal microorganisms and then vaccinated against *Salmonella* (Sm24/Rif12/Ssq strain) at days 2, 3, 4, and 5. The blood, liver, and ceca with contents were collected 5 days after immunization to isolate the *Salmonella* live attenuated vaccine strain. The number of *Salmonella* colonies in the blood, liver, and ceca with contents of each specific pathogen-free (SPF) chicken in the experimental group and the control group was calculated by the *Salmonella* selection medium, and the changes in the amount of *Salmonella* colonization in the blood, liver, and ceca with contents were measured. The aim was to elucidate the effects of different antibiotics and different withdrawal times on the colonization of the *Salmonella* vaccine in chickens. Timely withdrawal of these antibiotics is of paramount importance to set the primer for local and cellular immunity at an early age, enabling the prevention and control of *Salmonella* Enteritidis in the poultry industry.

## Materials and Methods

### Strains and Growth Conditions

For the detection of vaccine strains, Sm24/Rif12/Ssq modified Brilliant-Green Phenol-Red Lactose Sucrose (BPLS) agar (Beijing Land Bridge, Beijing, China) was used. The BPLS agar was supplemented with rifampicin at a concentration of 100 μg/ml and streptomycin at a concentration of 200 μg/ml. To prepare a 1% rifampicin (Sigma, St. Louis, MO, USA) stock solution, 1 g of rifampicin was dissolved in 100 ml DMSO (Sigma, St. Louis, MO, USA) by stirring and storing at room temperature while protected from light. To prepare streptomycin (Sigma, St. Louis, MO, USA) stock solution (1%), 1 g of streptomycin was dissolved in 100 ml Aqua Dest. To prepare rifampicin- and streptomycin-containing agar plates (100 μg/ml rifampicin and 200 μg/ml streptomycin), 5 ml of rifampicin and 10 ml of streptomycin were added to the stock solution of 500 ml and dissolved in hand-warm (50°C) BPLS agar.

### Animals

The SPF eggs were from Lihua (Lihua Agricultural Technology Co., Ltd., Zhejiang, China) and were incubated in a local *Salmonella*-free hatchery. Newly hatched day-old SPF White Leghorn chickens were housed in an SPF chicken isolator under a controlled temperature of 28–30°C with a 12-h light/dark cycle and free access to food and water during the study period.

### Experimental Groups and Treatments

A total of 240 1-day-old chicks were randomly divided into 24 groups of 10 animals. Following the instructions of the manufacturer, different groups were treated with different antibiotics, which included ceftiofur (the dose was 0.1 mg per chicken by intramuscular injection at 1 day old; the withdrawal time is 3 days; Zoetis, New Jersey, USA), amoxicillin (amoxicillin was administered by drinking water for 5 days; the dose was 10 g/L water, and the withdrawal time is 7 days; Merck, New Jersey, USA), enrofloxacin (enrofloxacin was administered by drinking water for 5 days; the dose was 0.75 ml/L water, and the withdrawal time is 8 days; Bayer, Leverkusen, Germany), lincomycin–spectinomycin (lincomycin–spectinomycin was administered by drinking water for 5 days; the dose was 150 mg/kg chicken weight, and the withdrawal time is 7 days; Zoetis, New Jersey, USA). The control group was not treated with antibiotics. Each group was immunized with the *Salmonella* Enteritidis live vaccine at 2, 3, 4, and 5 days after withdrawal of the antibiotics, respectively. Meanwhile, the control group was immunized without treatment. The detailed grouping information is shown in Table 1.

**Table 1:** Experimental design.

**Experimental groups**	**Antibiotic**	**Administration time**	**Vaccination time after withdrawal (day)**	**Bird age of vaccination (days old)**	**Bird age of recovered *Salmonella* (days old)**
1	Ceftiofur	1 day old	2	3	8
2	Ceftiofur		3	4	9
3	Ceftiofur		4	5	10
4	Ceftiofur		5	6	11
5	Sterile phosphate-buffered saline (PBS) instead		2	3	8
6	Sterile PBS instead		3	4	9
7	Sterile PBS instead		4	5	10
8	Sterile PBS instead		5	6	11
9	Amoxicillin	From 1 to 5 day old	2	7	12
10	Amoxicillin		3	8	13
11	Amoxicillin		4	9	14
12	Amoxicillin		5	10	15
13	Enrofloxacin		2	7	12
14	Enrofloxacin		3	8	13
15	Enrofloxacin		4	9	14
16	Enrofloxacin		5	10	15
17	Lincomycin–spectinomycin		2	7	12
18	Lincomycin–spectinomycin		3	8	13
19	Lincomycin–spectinomycin		4	9	14
20	Lincomycin–spectinomycin		5	10	15
21	None		2	7	12
22	None		3	8	13
23	None		4	9	14
24	None		5	10	15

### Vaccine and Vaccination

#### Vaccine Source

ELANCO *Salmonella* Enteritis live vaccine (Sm24/Rif12/Ssq strain, AviProTM *Salmonella* Vac E) was manufactured in Cuxhaven, Germany. Following the instructions of the manufacturer, the contents of the 2,000-dose vaccine vial were diluted in 1 L of sterile, pure water. The birds were individually vaccinated with 0.5 ml (1.1 × 10^8^ CFU) vaccine by oral gavage into the crop according to the vaccination schedule of each trial (Table 1).

### *Salmonella* Enteritidis Vaccine Strain Examination in vivo

At 5 days after immunization, the blood of the chickens were collected with an anticoagulant tube. Then, the chickens were humanely euthanized with CO_2_ in an inhalation chamber according to the approved protocol. Phosphate-buffered saline was used to homogenize or serially dilute chicken tissue samples from the blood, ceca with contents, and liver. The diluted samples were plated onto BPLS agar (rifampicin of 100 μg/ml and streptomycin of 200 μg/ml) and cultured at 37°C overnight, and the number of bacteria in each sample was counted using the plate counting method.

### *Salmonella* Enteritidis Vaccine Strain Examination by PCR

Buffered peptone water (BPW, Beijing Land Bridge, Beijing, China) was prepared according to the instructions of the manufacturer. The liver and blood from each bird were sampled and mixed with BPW (1:10) and incubated overnight (18–20 h) at 37°C. From each of these incubated tubes, 250 μl was transferred into another tube containing 5 ml BPW and incubated overnight at 37°C. Furthermore, the bacteria were boiled for 5 min to extract the genomic DNA for PCR detection of *Salmonella* (15). The sequences of the PCR primer sequence are salm-invA-F (GGAACGAACTAATTCAGCGATA) and salm-invA-R (AGATGTCATTAACCTTGTGGAG). The product size is 435 bp. Meanwhile, the colonies of ceca with contents on the BPLS plate were examined by PCR.

### Statistical Analysis

Statistical analyses were conducted using SPSS V19.0 software (SPSS Inc., Chicago, IL, USA). Student's *t*-test was used to analyze the data, and *p*-values <0.05 were considered significant.

## Results

### Results of *Salmonella* Isolation and Counting in the Blood

**Figure 1:**
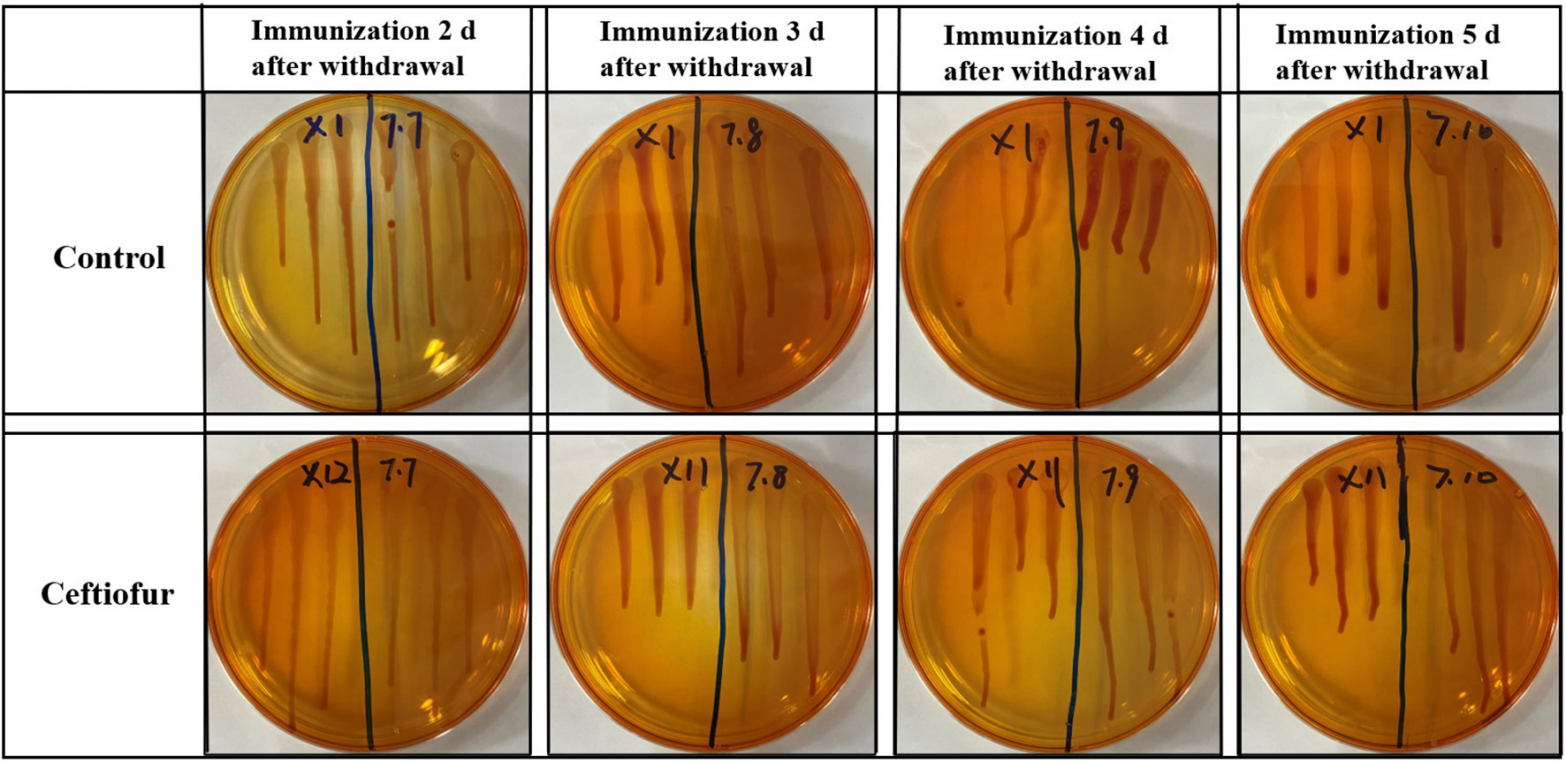
Detection of *Salmonella* vaccine strains in blood from ceftiofur groups. No matter how many days after withdrawal the chicks were immunized, no *Salmonella* vaccine strain was detected in the blood of chickens in both the ceftiofur and control groups.

The ceftiofur group was vaccinated with *Salmonella* vaccine on the second, third, fourth, and fifth day after withdrawal, respectively. At 5 days after immunization, the blood of SPF chickens was collected from the ceftiofur and the control groups. The *Salmonella* vaccine strain in the original blood was isolated and counted by using *Salmonella* identification medium BPLS medium (containing 100 μg/ml rifampicin and 200 μg/ml streptomycin). The count of *Salmonella* in the blood solution is shown in Figure 1, and no colony formation was isolated from the blood. The same method was used to isolate the *Salmonella* vaccine strain in the original blood of the amoxicillin, enrofloxacin, and lincomycin–spectinomycin groups, and no *Salmonella* vaccine strain was isolated in the blood of these groups (Supplementary Figure S1).

**Figure 2:**
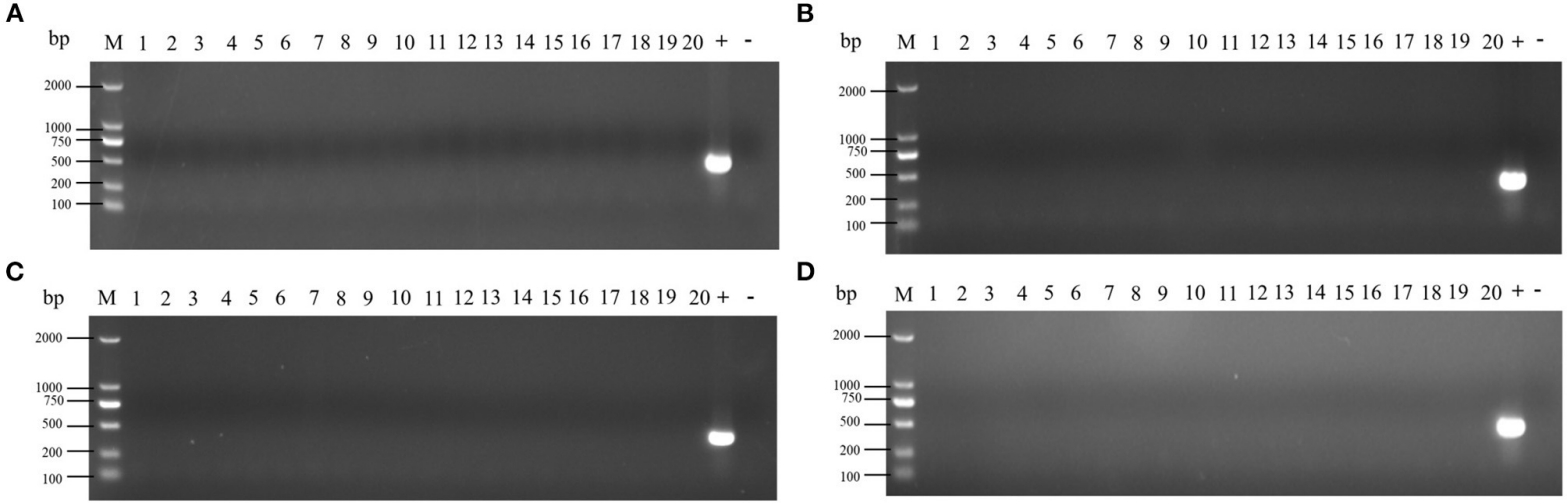
Detection of *Salmonella* in the blood by PCR from ceftiofur groups. **(A)** Immunization 2 days after withdrawal: 1–10, control group; 11–20, ceftiofur group; +, positive control; –, negative control. **(B)** Immunization 3 days after withdrawal: 1–10, control group; 11–20, ceftiofur group; +, positive control; –, negative control. **(C)** Immunization 4 days after withdrawal: 1–10, control group; 11–20, ceftiofur group; +, positive control; –, negative control. **(D)** Immunization 5 days after withdrawal: 1–10, control group; 11–20, ceftiofur group; +, positive control; –, negative control.

After the blood was enriched by BPW, DNA was extracted from the enrichment broth, and PCR detection was performed with *Salmonella*-specific primers. The PCR test results of all blood samples were negative in the ceftiofur group (Figure 2). The other experimental groups (amoxicillin, enrofloxacin, and lincomycin–spectinomycin groups) did not detect *Salmonella* in the blood by PCR (Supplementary Figure S2). The above-mentioned results showed that no *Salmonella* vaccine strain (Sm24/Rif12/Ssq strain) was isolated from the blood of the experimental group and the control group.

### Results of *Salmonella* Isolation and Counting in the Liver

At 5 days after immunization, the liver of SPF chickens were collected in the experimental group and the control group. The *Salmonella* vaccine strain in liver homogenate was isolated and counted on BPLS agar plates (containing 100 μg/ml rifampicin and 200 μg/ml streptomycin). The count of *Salmonella* in liver homogenate is shown in the supplementary material (see Supplementary Figure S3 for the ceftiofur group; other experimental groups are shown in Supplementary Figure S4), no colony formation was isolated in liver homogenate on BPLS medium at 2–5 days post-withdrawal.

After the liver homogenate was enriched in BPW, DNA was extracted from the enrichment broth, and PCR detection was performed with *Salmonella*-specific primers. The PCR test results of all liver homogenate samples were negative (shown in Supplementary Figure S5). The abovementioned results showed that no *Salmonella* vaccine strain was isolated from a liver homogenate of the experimental group and the control group.

### Results of Isolation and Counting of *Salmonella* in the Cecum

**Figure 3:**
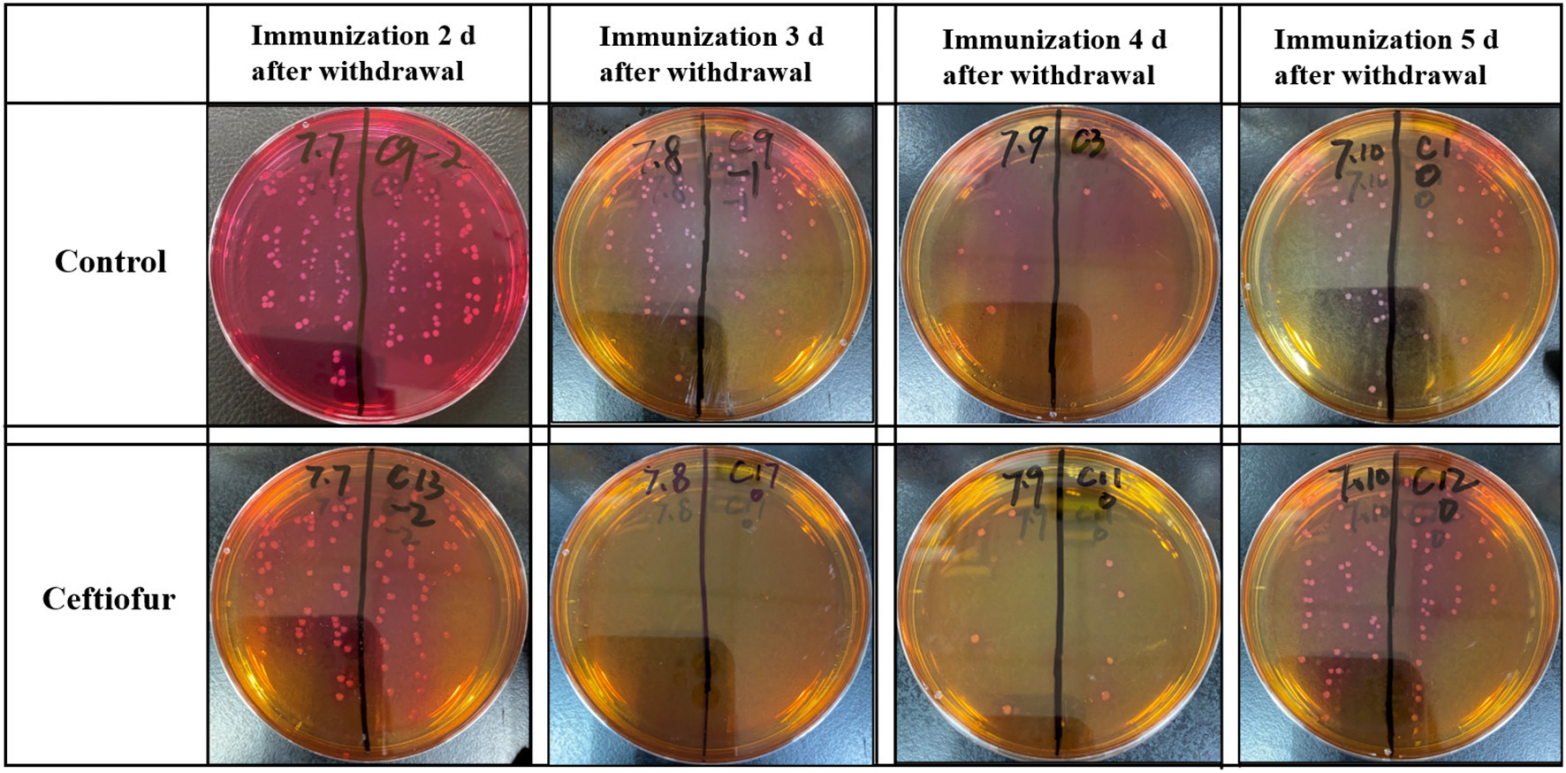
Isolation of *Salmonella* from cecum after from the ceftiofur groups. No *Salmonella* vaccine strains were isolated from the cecum of chickens immunized at 3 days after ceftiofur withdrawal. In chicks immunized at other times after withdrawal, the *Salmonella* vaccine strain could be isolated from the cecum. The *Salmonella* vaccine strain appears as reddish smooth colonies on Brilliant-Green Phenol-Red Lactose Sucrose medium.

**Figure 4:**
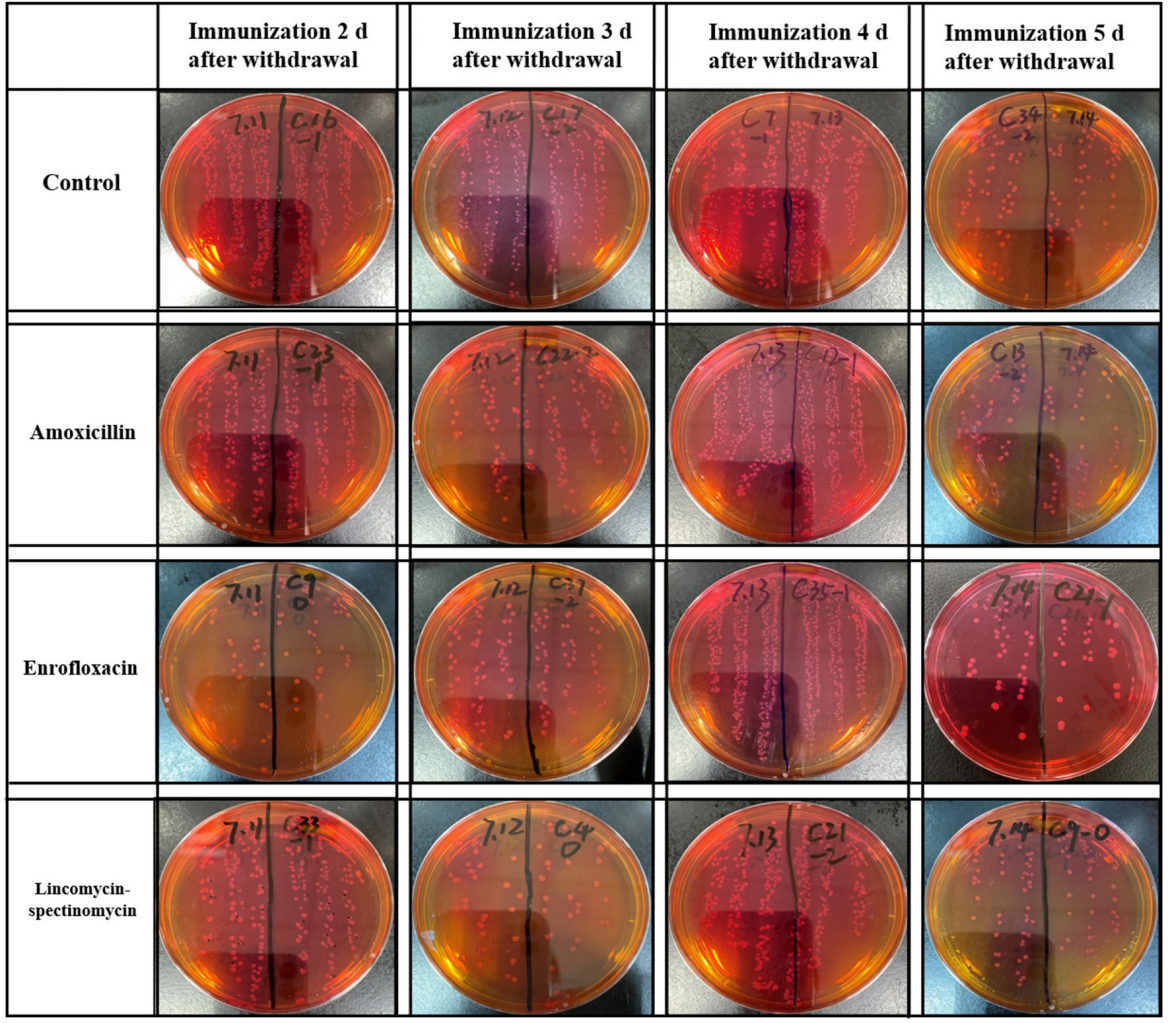
Isolation of *Salmonella* from cecum in the amoxicillin, enrofloxacin, and lincomycin–spectinomycin groups. No matter how many days after withdrawal of antibiotics the chicks were immunized, the *Salmonella* vaccine strains isolated in the cecum of chicks were reddish smooth colonies on Brilliant-Green Phenol-Red Lactose Sucrose medium.

At 5 days after immunization, the ceca with contents of SPF chickens were collected in the experimental group and the control groups. The cecum plus content homogenate were isolated, and colonies were counted on BPLS agar plates (containing 100 μg/ml rifampicin and 200 μg/ml streptomycin). The colony formation of *Salmonella* in the ceca with content homogenate in the ceftiofur group is shown in Figure 3. The colony counts of the amoxicillin, enrofloxacin, and lincomycin–spectinomycin groups are shown in Figure 4. *Salmonella* vaccine strains appear as reddish smooth colonies on BPLS medium. The suspected colonies on the BPLS medium were detected by PCR with *Salmonella*-specific primers, and the bacteria produced amplicons with the bands consistent with the expected size (data not shown).

**Figure 5:**
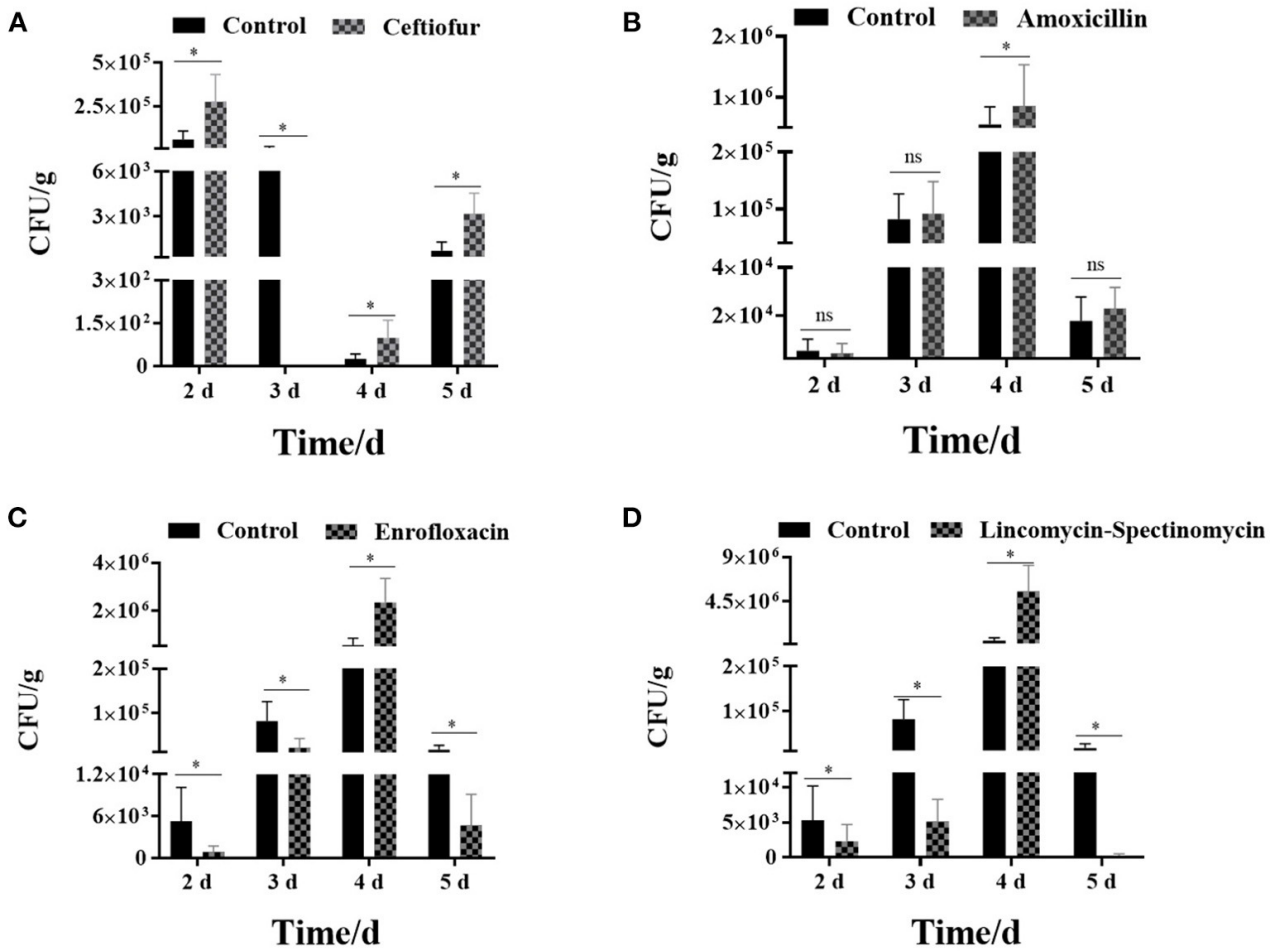
Count of *Salmonella* vaccine strains in the cecum from various groups. **(A)** The number of *Salmonella* vaccine strains colonized in the cecum in the ceftiofur group. **(B)** The number of *Salmonella* vaccine strains colonized in the cecum in the amoxicillin group. **(C)** The number of *Salmonella* vaccine strains colonized in the cecum in the enrofloxacin group. **(D)** The number of *Salmonella* vaccine strains colonized in the cecum in the lincomycin-spectinomycin group. Differences between mean values were assessed by an unpaired two-tailed Student's *t*-test (ns, non-significant; *P < 0.05 represents a significant difference in numbers between the two groups).

The results showed that the number of *Salmonella* in the cecum was higher in the ceftiofur group than in the control group, except for immunization at 3 days after the withdrawal of antibiotics (*P* < 0.05) (see Figure 5A). Most *Salmonella* vaccine strains were detected during immunization 2 days following ceftiofur withdrawal, while it was not isolated in the ceftiofur group at 3 days after withdrawal. The number of *Salmonella* cecum colonization was highest in the three experimental groups (amoxicillin, enrofloxacin, and lincomycin–spectinomycin groups) among the chickens immunized at 4 days after withdrawal, which was higher than in the other withdrawal intervals (2, 3, and 5 days) and higher than in the control group (*P* < 0.05) (see Figures 5B–D). However, immunization at other withdrawal intervals (2, 3, and 5 days) did not affect the colonization of *Salmonella* vaccine strains in cecum in the amoxicillin group (*P* > 0.05) (see Figure 5B). Furthermore, at different stages of withdrawal, the amount of cecal *Salmonella* vaccine strain in the immunized chicks was higher in the control group than in the enrofloxacin and the lincomycin–spectinomycin groups (*P* < 0.05).

## Discussion

*Salmonella* control in poultry farms is more than ever a critical issue. According to annual reports from the European Union (EU), the United States, and Brazil, consuming contaminated food is the leading cause of *Salmonella* infection in humans (16, 17). Each year, the Centers for Disease Control and Prevention reports 40,000 human cases of salmonellosis in the United States. More than 160,000 cases of salmonellosis were recorded in the EU in 2006, resulting in an annual incidence of 34.6 cases per 100,000 people (16). The European Food Safety Agency report showed that salmonellosis was the second most reported zoonotic disease in the EU in 2019, affecting about 88,000 people.

Due to multiple routes of transmission, *Salmonella* may easily enter the food chain and cause human enteritis. Salmonellosis can be serious in high-risk groups such as infants, young children, and the elderly. Therefore, the primary goal of controlling *Salmonella* in poultry is to tackle the infection in primary production to systematically reduce the contamination of poultry products and then, ultimately, the spread to humans. Experts focused on the risk to consumers posed by *Salmonella* Enteritidis, the bacterium responsible for causing the highest number of egg-borne outbreaks in the EU. At the same time, the protection of *Salmonella* Typhimurium from very young chicks may lead to serious diseases and huge economic losses in the early stages of life (18).

At present, vaccine programs based on inactivated vaccines and live vaccines have been used to control *Salmonella* (19). The inactivated vaccine can stimulate the production of antibodies but does not affect the proliferation of immune system cells (20). Live vaccines can induce strong humoral and cellular immune responses, especially when the vaccine strains are invasive and systematically present all their antigens (21). After a robust vaccination program, the immunity of chicks infected with *Salmonella* to reinfection was significantly improved. In general, live vaccines are considered more suitable for stimulating cellular immunity and have a protective effect on poultry products (18, 22). Some live vaccines thus achieve a double mechanism of protection by increasing mucosal immunity and producing specific bacterial competition against any wild-type strain of *Salmonella* (23, 24). The two mechanisms work together to significantly reduce colonization and excretion, which can only be successfully achieved by oral vaccination in the early colonization stage of infection (22). Nowadays, under the condition of intensive breeding in the world, young birds are prone to stress syndrome in the brooding stage, which is mainly caused by inflammation, digestive dysfunction, exotic pathogen infection, microbial flora imbalance, and diarrhea (25, 26). Hence, the responsible use of antibiotics is necessary to prevent and treat diseases as well as to promote growth but, at the same time, be cautious about the risk of antimicrobial resistance (27). In multiple geographies, poultry flocks are often raised under intensive conditions using large amounts of antimicrobials to prevent and treat disease and to promote promotion. The aim of this study was to understand whether the use of antibiotics affects the colonization of the *Salmonella* live attenuated vaccine in chickens after oral administration. We investigated four antibiotics that are the most commonly used on chickens in poultry farms and tested the interference of these four antibiotics on the colonization of the *Salmonella* Enteritidis live vaccine (Sm24/Rif12/Ssq strain). Our experimental results showed that no live vaccine strain was isolated from the blood and liver; in contrast, the *Salmonella* Enteritidis live vaccine strain was consistently isolated from the cecum. This finding is consistent with previous studies, which also have shown that it is not common to isolate the live *Salmonella* vaccine strain from livers, but that it is easier to recover the live vaccine strain from the cecum (19). Accordingly, our study has shown that no *Salmonella* Enteritidis live vaccine strain was isolated from the blood and liver of the chicks. The reason for that could be that the *Salmonella* live vaccine strain is attenuated under the metabolic drift mutation principle, which confers limited persistence in internal organs and limited shedding (28). It also shows that this vaccine is safe to use.

In this study, we found that the number of organisms of the *Salmonella* live vaccine strain in the cecum was higher in the ceftiofur group than in the control group, except for immunization at 3 days after withdrawal. Ceftiofur is a 3GC approved in various countries worldwide to control the early mortality associated with bacterial infections in poultry, being only registered for veterinary use. The mechanism of ceftiofur is to destroy the transcriptase peptidase and block the synthesis of mucopeptides so that the bacterial cell wall fails to achieve the bactericidal effect (29). Ceftiofur is a broad-spectrum antibiotic, and it has a strong antibacterial effect on both gram-positive and gram-negative bacteria. The use of ceftiofur can cause imbalances in the bacterial populations, but due to systemic administration, it has a limited compromising effect on the colonization of *Salmonella* live vaccine in the cecum (30). Following the corresponding testing procedure in the lab, the *Salmonella* live vaccine strain was not detected in the ceftiofur group at 3 days after withdrawal; ceftiofur did show a strong effect at this time, killing the *Salmonella* live vaccine strain and preventing the vaccine strain from colonizing the cecum. In previous studies, the mode of action of both products was properly documented; live vaccines are attenuated strains that can very well colonize the intestines, while antibiotics are used to neutralize or inhibit bacteria. Therefore, there is a probability for antimicrobials to greatly reduce gut colonization by the live vaccines (31). Following immunization at 4 days after withdrawal, the amount of colonizing *Salmonella* live vaccine strain in the cecum of chicks was very low in both the ceftiofur and control groups, possibly because the normal intestinal flora of chicks changed during this period, which is not favorable to the colonization of *Salmonella* vaccine.

Amoxicillin, an antibiotic with a broad spectrum of action, belongs to the class of penicillins and the subclass of aminopenicillins. All β-lactam antibiotics are bactericidal by interfering with the biosynthesis of peptidoglycan, a component of the bacterial cell wall. When administered orally, amoxicillin remains stable in an acidic medium; it is well-absorbed by the gastrointestinal tract and has good tissue penetration (12). With respect to enrofloxacin, this is a quinolone developed specifically for animal use and has a broad antibacterial spectrum, with a potent effect against a wide range of gram-positive and gram-negative bacteria. It is one of the most widely used antibiotics in veterinary medicine for the treatment and prevention of diseases, having a direct neutralizing effect by inhibiting bacterial DNA-gyrase and topoisomerase IV enzyme activities (13). It has also been documented that fluoroquinolone treatment can affect the intestinal microbiota (32). Regarding lincomycin and spectinomycin, both are antibiotics that affect the translational machinery in the target bacteria. Lincomycin inhibits protein synthesis in sensitive bacterial strains by blocking the peptidyltransferase process by interacting with both the A-site and the P-site on the 50 S ribosomal subunit, affecting the placement of both tRNA molecules and directly inhibiting peptide bond formation (33, 34). Spectinomycin (closely related to aminoglycoside), on the other hand, is an aminocyclitol antibiotic that binds to helix-34 inside the 30 S ribosomal subunit to impede translocation and protein synthesis (35). Lincomycin–spectinomycin, as a substitute for ceftiofur, also has broad-spectrum antibacterial properties. Most anaerobic bacteria, both gram-positive and gram-negative, are sensitive to lincomycin. Specifically, the populations of *Enterococcus* and *Lactobacillus* were shown to be significantly reduced by lincomycin (36). Lincomycin–spectinomycin are broad-spectrum antibacterial agents indicated to treat infections of intestinal and respiratory tracts in poultry caused by *S*. Enteritidis, *Avibacterium gallinarum, Escherichia coli, Mycoplasma synoviae, Mycoplasma gallisepticum*, and *P. multocida* (37). Apart from their therapeutic properties, antibiotics have also been shown to disrupt the “normal” composition of microbiota and used to study the development of the intestinal immune system at the same time. Antibiotics, as previously reported, can transiently alter the microbiota, both in terms of cecal microbiota diversity and the composition of microbial groups found in the jejunum and the cecum (38). The number of *Salmonella* live vaccine strain cecum colonization in the three experimental groups (amoxicillin, enrofloxacin, and lincomycin–spectinomycin groups) was higher than in the control group among chickens immunized at 4 days after withdrawal. The quantity of *Enterococcus* and *Lactobacillus* in the chick intestine was dramatically reduced as a result of antibiotic treatment, making the environment more favorable to the colonization of the *Salmonella* live vaccine strain.

On days 2 and 3 after withdrawal, the amount of *Salmonella* live vaccine strain in the cecum of chicks in the enrofloxacin and lincomycin–spectinomycin groups was not as high as that in the control group. The reason may be that enrofloxacin and lincomycin–spectinomycin have such a strong effect at this time, killing the *Salmonella* vaccine strain and preventing it from colonizing the cecum.

The amount of *Salmonella* live vaccine strain in the cecum of chicks in the enrofloxacin and lincomycin–spectinomycin groups was likewise not as high as in the control group at 5 days after withdrawal. According to previous research reports, some significant changes at 5 days after antibiotic administration were seen in the intestinal immunological development as a response probably associated with a change in microbiota because cytokine mRNA expression in the chick intestinal tract was either up- or downregulated. Even though the microbiota, when exposed to several antibiotic groups, can be altered, certain bacterial constellations can also trigger an impact on the immune system (38). It is possible that changes in the immune system are not favorable to *Salmonella* live vaccine colonization in the cecum. Therefore, the isolation rate of the experimental group was lower than that of the control group. Antibiotics transiently changed the microbiota in different organs, both with respect to the diversity of cecal microbiota as well as the composition of microbial groups occurring in the jejunum and the cecum (38). Because these three organs are adjacent to each other and the microorganisms in each organ are different, the use of antibiotics will cause the transfer of microorganisms among each of the three organs, which will affect the colonization of the *Salmonella* vaccine in the cecum. Furthermore, the different routes of administration may also affect the colonization of *Salmonella*. The possible reason is that antibiotics administered by different routes take a different time to exert their effects. Ceftiofur can quickly function and change the gut microbiota by intramuscular injection, which is more conducive to the colonization of *Salmonella* vaccine. However, the antibiotics through drinking water are not as effective as the intramuscular injections. Because the concentration of antibiotics in the intestine is relatively low, it takes longer to affect the changes in the gut microbiota.

Considering the fact that antibiotics and *Salmonella* vaccination are two of the most commonly utilized interventions in poultry production, the results documented from this study are expected to have significant clinical and public health implications. These findings can potentially be used to enable good vaccination practices in the field while providing guidance for future studies into approaches to better understand the interaction and balance between the gut microbiota, responsible medication with antibiotics, and the immune response derived from *Salmonella* live vaccines.

## Conclusion

The research conducted throughout this study can assist poultry managers and veterinarians to determine the best timing to deploy a *Salmonella* live vaccine in poultry flocks after antibiotic treatment. When the chicks are exposed to ceftiofur medication applied by intramuscular route, it is then recommended to wait for at least until day 4 after withdrawal to conduct the oral *Salmonella* live vaccination in order to minimize the risk for the vaccine strain to be neutralized by effective concentrations of this broad-spectrum third-generation cephalosporin which might still be circulating across different tissues. When the chicks are orally treated *via* drinking water with amoxicillin, enrofloxacin, or lincomycin–spectinomycin, then the minimum withdrawal time should be 3 days following the last antibiotic dose before immunization since this is more favorable to *Salmonella* Enteritidis vaccine colonization, which was mostly associated with the cecum. No *Salmonella* vaccine strains were isolated from the blood or liver, indicating that the attenuated *Salmonella* live vaccine strain has a reduced organ invasion pattern and limited systemic dissemination. Thus, the vaccine dose was duly considered safe for those immunized animals.

## Data Availability Statement

The original contributions presented in the study are included in the article/Supplementary Material, further inquiries can be directed to the corresponding author.

## Ethics Statement

The care and maintenance of all animals were performed following the guidelines of the Institutional Animal Care and Use Committee of Shanghai Veterinary Research Institute, Chinese Academy of Agricultural Sciences (CAAS). The Ethics Committee of CAAS approved the use of chicks for this study. The permit was documented under the number SHVRI-SD-20200623-03.

## Author Contributions

JH and XH designed the experiment. JH, CC, JZ, XN, ZW, LL, YJ, HZ, and TZ were involved in the acquisition of the experimental data. The main work of JH, CC, JZ, and XN were raising chickens and immunizing animals. The *Salmonella* vaccine strains were counted by ZW, LL, YJ, HZ, and TZ. JH, CC, XH, XN, and FY performed data analysis and interpretation. The manuscript was drafted and revised for important intellectual content by JH, CC, JZ, XN, SN, and XH, as well as final approval of the version to be published with agreement to be accountable for all aspects of the work in ensuring that questions related to the accuracy or integrity of any part of the work are appropriately investigated and resolved. All authors contributed to the article and approved the submitted version.

## Funding

This work was supported by the fund of the National Natural Science Foundation of China (31872483 and 32072829) and Experimental Animal of Shanghai Science and Technology Committee (grant/award number: 18140900700).

## Conflict of Interest

The authors declare that they have no conflicts of interest to this work. We declare that we do not have any commercial or associative interest that represents a conflict of interest in connection with the work submitted.

## Publisher's Note

All claims expressed in this article are solely those of the authors and do not necessarily represent those of their affiliated organizations, or those of the publisher, the editors and the reviewers. Any product that may be evaluated in this article, or claim that may be made by its manufacturer, is not guaranteed or endorsed by the publisher.
