# Supplementary material for: Effect of Antibiotics on the Colonization of Live Attenuated Salmonella Enteritidis Vaccine in Chickens
Source: Front Vet Sci. 2021 Dec 1;8:784160. doi: 10.3389/fvets.2021.784160 (PMC8671454; doi:10.3389/fvets.2021.784160)
Supplement: Supplementary file 1 [file Data_Sheet_1.PDF]

Bacteriological culture method for detection of SE (Sm24/Rif12/Ssq) and ST (Nal2/Rif9/Rtt) vaccine strains from environment, poultry specimens and drinking water

OVERVIEW

For accurate detection of SE (Sm24/Rif12/Ssq) and ST (Nal2/Rif9/Rtt) vaccine strains, the following standardised testing procedures have demonstrated to be sensitive, simple and reliable. The given technical steps are intended for routine implementation at poultry diagnostic laboratories in order to assure international acceptance and consistent interpretation of test results when detecting vaccine strains contained in AviPro™ Salmonella Vac E, AviPro™ Salmonella Vac T and AviPro™ Salmonella DUO.

Specimen (WHERE)	Sample (WHAT)	Timing of sampling (WHEN)	Approach (HOW)	Rational (WHY)
**ENVIRONMENT**	**Sock swabs** TechNote #001	**2 days** post-vaccination	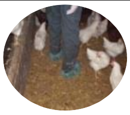 Pool of 20 samples in each house x 5.	POST-take assessment of vaccine intake (SE+ST) after 1st vaccination
	**Litter** TechNote #001	**2 days** post-vaccination		Assess shedding pattern of vaccine strain
	**Water lines** TechNote #002	During vaccination	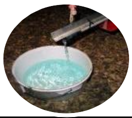 Direct collection with BPLS plates (supplemented with Antibiotic markers)	Verify correct posology
**BIRDS**	**Cloacal swabs** TechNote #001	**4-5 days** post-vaccination	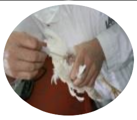 Total of 100 birds (grouped in 5 pools, each pool having 20 birds)	Assess colonization of intestine and reproductive tract
	**Liver & Spleen** TechNote #001	**5 days** post-vaccination **IMPORTANT:** Day 5 is the best time to do the organ sampling as it is the time when it is easier to recover both SE and ST vaccine strains.	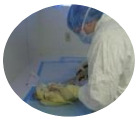 Birds are euthanized and samples (pooled liver/spleen) are aseptically collected and placed into individual re-sealable plastic. **Sample size of 20 birds (grouped in 4 pools, each pool having 5 birds)**	Assess organ invasion
	**Ceca plus content** TechNote #001	**7 days** post-vaccination	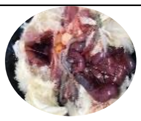 Birds are euthanized and samples (pooled intestinal tract with contents, including ceca with contents) are aseptically collected and placed into individual re-sealable plastic bags. **Sample size of 20 birds (grouped in 4 pools, each pool having 5 birds)**	Assess ceca colonization<br><br>Pooled whole intestinal tract with contents (IT), and ceca with tonsils and contents (CC)

TechNote #001 (*POST-take*): Detection in environmental and poultry specimens

PURPOSE

Detect the presence of SE (Sm24/Rif12/Ssq) and ST (Nal2/Rif9/Rtt) strains contained in AviPro™ Salmonella Vac E, AviPro™ Salmonella Vac T and AviPro™ Salmonella DUO, from environmental and poultry specimens, to assure appropriate vaccine intake.

SAMPLING

The table above summarizes the approach for sample collection from vaccinated birds. Key considerations:

- Vaccine detection is recommended after the first dose only; this is due to the fact that the shedding of the MDM vaccine strain is substantially decreased with subsequent booster vaccinations.
- SE (Sm24/Rif12/Ssq) has a shorter shedding pattern than ST (Nal2/Rif9/Rtt) vaccine strain.
- Protection can only be tested through challenge infection with virulent *Salmonella*, in terms of LOG reduction at specific time points.
- Out of all matrices mentioned above, organ samples tend to have a higher recovery rate.
- Samples should be shipped to the laboratory in a foam cooler packaged for overnight express delivery (see packaging and shipping instructions on the next page).

PACKAGING AND SHIPPING SAMPLES TO LABORATORY

1. Use sterile techniques when removing tissues from birds to prevent cross-contamination of the samples. Change gloves and rinse tools in 70% ethanol between birds.
2. Organs to be sampled from each bird in the following order (sample volume not to exceed 3g/bag):
   a. Approximately 1-2g of pooled spleen and liver (LS) are placed in one small plastic re-sealable or Whirl-Pak™ bag.
   b. Approximately 1-2g of pooled intestinal tract with contents (IT), and ceca with tonsils and contents (CC) will be placed in a one small plastic re-sealable or Whirl-Pak™ bag.
3. Use a permanent marker, label re-sealable plastic or Whirl-Pak™ bags with bird number, flock number and date of collection.
4. There should be two bags of pooled tissues (liver/spleen vs. ceca plus content) per house.
5. Complete a laboratory submission form listing the bird number, sample type, farm name, house number and vaccine used.
6. Place samples and submission form in a polystyrene foam cooler with frozen cold packs and place in a fiberboard box.
7. Ship package of samples to an accredited-partner laboratory for next day express delivery. Transportation needs to take place as soon as possible, otherwise *Salmonella* bacteria can be compromised (overgrown by other organisms). For overnight storage is necessary to preserve the sample (pre-enriched in BPW) under fridge/refrigerator temperatures (e.g. between 2-6 ºC) – DO NOT FREEZE. Contact your Elanco sales representative or technical consultant for specific laboratory information.

WORKFLOW

1. **Pre-enrichment**

   Buffered Peptone Water (BPW) should be prepared according to manufacturer's instructions. One part of sample material is mixed with nine parts BPW (1:10) and incubated overnight (18-20 hrs) at 37°C. One swab should be enriched with 9 ml BPW, 10 swabs with 90 ml BPW, respectively.

2. **Isolation of colonies**

   After enrichment of *Salmonella*, a loopful of the BPW is streaked onto an appropriate selective plating media. These media are incubated overnight (18-20 hrs) at 37°C.

   - Selective plating media: Single application of AviPro™ Salmonella Vac T or AviPro™ Salmonella Vac E: For detection of either AviPro™ Salmonella Vac T or AviPro™ Salmonella Vac E modified Brilliant-green Phenolred Lactose Sucrose (BPLS) agar (e.g. Merck, cat. no. 1.10747) is used. The BPLS agar should be supplemented with Rifampicin[1] at concentration of 100 µg /ml.
   - Combined Application of AviPro™ Salmonella Vac T and AviPro™ Salmonella Vac E: For detection of AviPro™ Salmonella Vac T and AviPro™ Salmonella Vac E two BPLS agar plates have to be used in parallel. For detection of AviPro™ Salmonella vac T, one BPLS agar plate should be supplemented with Rifampicin at concentration of 100 µg /ml and Nalidixic acid[2] at concentration of 5 µg/ml. For the detection of AviPro™ Salmonella Vac E, another BPLS agar plate should be supplemented with Rifampicin at concentration of 100 µg /ml and Streptomycin[3] at concentration of 200 µg /ml.

[1]To prepare Rifampicin (Sigma, cat. no. R3501) stock solution (1%) dissolve 1 g of Rifampicin in 100 ml DMSO (Sigma, cat. no. D5879) under stirring and store at room temperature protected from light. The shelf live is three months. To prepare Rifampicin-containing agar plates (100 µg /ml Rifampicin) add 5 ml of Rifampicin stock solution to 500 ml dissolved hand-warm (50°C) BPLS agar plates. Store at 2°-8°C for not longer than two weeks.

[2]To prepare Nalidixic acid (Sigma, cat. no. N5035) stock solution (1%) dissolve 1 g of Nalidixic acid in 6 ml 1n NaOH and add 94 ml aqua dest. and store at 2°-8°C. The shelf live is three months. To prepare Rifampicin- and Nalidixic acid-containing agar plates (100 µg /ml Rifampicin and 5 µg /ml Nalidixic acid) add 5 ml of Rifampicin and 0.25 ml of Nalidixic acid stock solution to 500 ml of dissolved hand-warm (50˚C) BPLS agar. Store at temperature below 15°C at 2°-8°C for not longer than two weeks.

[3]To prepare Streptomycin (Sigma, cat. no. S9137) stock solution (1%) dissolve 1 g of Streptomycin in100 ml aqua dest. The shelf life is three month. To prepare Rifampicin- and Streptomycin-containing agar plates (100 µg /ml Rifampicin and 200 µg /ml Streptomycin) add 5 ml of Rifampicin and 10 ml of Streptomycin stock solution to 500 ml dissolved hand-warm (50°C) BPLS agar. Store at 2°-8°C for not longer than two weeks.

Table I. Antimicrobial agents used as resistance markers to discriminate field strains from vaccine strains.

		INTERPRETATION			
**Antibiotic**	**Concentration µg/ml**	**SE vaccine strain** (Sm24/Rif12/Ssq)	**SE Field strain**	**ST vaccine strain** (Nal2/Rif9/Rtt)	**ST Field strain**
Erythromycin (ERYD)	**20** µg/ml	**Sensitive** (none or very little growth)	Resistant	**Sensitive** (none or very little growth)	Resistant
Rifampicin (RAM)	**100** µg/ml	**Resistant** (evident growth)	Sensitive	**Resistant** (evident growth)	Sensitive
Streptomycin (STRE)	**200** µg/ml	**Resistant** (evident growth)	Sensitive	**Sensitive** (none or very little growth)	Sensitive
Nalidixic Acid (NAL)	**5** µg/ml	**Sensitive** (none or very little growth)	Sensitive	**Resistant** (evident growth)	Sensitive

- Results: Separate BPLS vaccine markers having individual vaccine strains inoculated do show specific growth pattern. It can be assumed that growth of bacteria on the antibiotic-containing BPLS agar plates will show only the SE vaccine strain (Rif12/Sm24/Ssq) or ST vaccine strain (Rif9/Nal2/Rtt) respectively. Agglutination with Somatic Antigens (O:9 for SE and O:4,5 for ST) can confirm this assumption.

*VacE agar: growth of RIF **100 ug/ml** & STR **200 ug/ml**

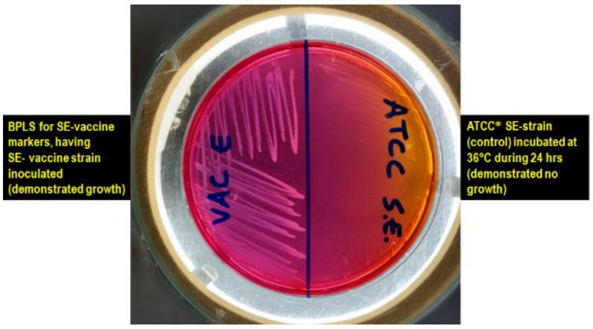

*VacT agar: growth of RIF **100 ug/ml** or NAL **5 ug/ml**

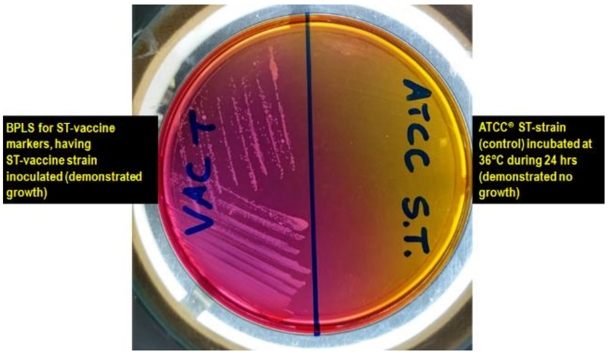

- Confirmation: SE (Sm24/Rif12/Ssq) and ST (Nal2/Rif9/Rtt) vaccine strains isolates may be confirmed by testing the susceptibility against Erythromycin using Mueller-Hinton agar with paper discs impregnated with 20 µg of Erythromycin.

3. Biochemical determination of genus

A positive *Salmonella*-result (both field and vaccine strain) can be biochemically confirmed using the API 20E test produced by BioMerieux (see manufacturer instructions).

4. Serotyping

Salmonella-like colonies are tested by means of rapid plate agglutination method. The serovar *S*. Enteritidis can be identified using the antisera O:**9** and H:gm. The serovar *S*. Typhimurium can be identified using the antisera O:**4,5** and H:i & H:1,2

5. Identification of AviPro™ Salmonella Vac E and AviPro™ Salmonella Vac T vaccine strains

Identification of SE strain (Rif12/Sm24/Ssq) or ST strain (Rif9/Nal2/Rtt) can be done by microdilution method using the AviPro™ PLATE, as described in the instruction manual supplied by MERLIN Diagnostika GmbH. In the absence of Nalidixic Acid, Streptomycin at the given concentration (200 µg/ml) enables the discrimination between both vaccine strains: SE strain (Rif12/Sm24/Ssq) is resistant to STRE while the ST strain (Rif9/Nal2/Rtt) is sensitive to STRE.

SUPPLIES

Selective Media and Antibiotics to be ordered directly to Merck KGaA-Sigma-Aldrich (now MilliporeSigma), requesting the specific catalogue numbers:

1. BPLS → Merck – Catalogue nr. 110747
2. Rifampicin → Sigma, Catalogue nr. R3501, diluted in DMSO (Dimetilsulfóxido)
3. Streptomycin 1% → Sigma, Catalogue nr. S9137, diluted in distilled water
4. Nalidixic Acid → Sigma, Catalogue nr. 29971 or nr. N8878-5G
5. DMSO (Dimethylsulfoxid) → Merck KGaA, Catalogue nr. 802912

TechNote #002 (*PRE-take*): Detection in drinking water

PURPOSE

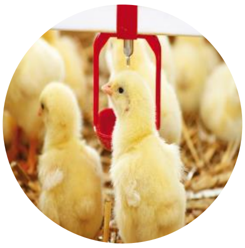

Detect the presence of SE (Sm24/Rif12/Ssq) and ST (Nal2/Rif9/Rtt) strains contained in AviPro™ Salmonella Vac E, AviPro™ Salmonella Vac T and AviPro™ Salmonella DUO, from drinking water, to assure appropriate vaccination procedure.

WORKFLOW

1. **Disposables, chemicals**
   - Containers for collection of vaccine water (plastic bottles, disposable plastic cups etc). They must not contain any residues of disinfectants or detergents. Use one vial per vaccine water sample.
   - For detection of AviPro™ Salmonella Vac T, use brilliant green phenolred lactose saccharose (BPLS) agar plate supplemented with Nalidixic acid[1] at concentration of 5 µg/ml and Rifampicin[2] at concentration of 100 µg/ml. Use one BPLS-agar plate per water sample.
   - For detection of AviPro™ Salmonella Vac E only, BPLS should be supplemented with Streptomycin[3] at concentration of 200 µg/ml. Use one BPLS-agar plate per water sample.
   - Use a sterile loop for streaking the sample (1 loop per vaccine water sample).

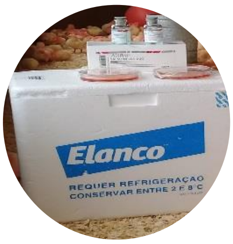

2. **Collection of water**

   The technical procedure to collect water sample simply depends on the type of drinking system in place. During the vaccination procedure, two samples are taken directly from the water tank and two samples from the water line at the end of the pipe: each vial should be identified by e.g. house, date, and sample location. A volume of **0.2 ml** of vaccine water should be collected from each sample location. The water sample should be free flowing thus avoiding any contact of the vial with the nipple or cup. Sterility of the water sample is not required. For each sample, a separate vial should be used.

3. **Sample Analysis**

   For detection of SE (Sm24/Rif12/Ssq) and ST (Nal2/Rif9/Rtt) strains then a loopfull vaccine water sample is streaked onto two separate BPLS-plates, each one supplemented with both vaccine markers, immediately after sample collection. This should be done ideally at flock side. For detection of SE (Sm24/Rif12/Ssq) only, a loopfull vaccine water sample is streaked onto BPLS-plates supplemented with Streptomycin & Rifampicin immediately after sample collection. For detection of ST (Nal2/Rif9/Rtt) strain only, a loopfull vaccine water sample is streaked onto BPLS-plates supplemented with Nalidixic Acid & Rifampicin immediately after sample collection.

   [1]To prepare Nalidixic acid (Sigma, Catalogue nr. 29971 or nr. N8878-5G) at 1% stock solution, dissolve 1g in 6ml 1n NaOH and add 94ml aqua dest. and store at 2°-8°C. The shelf live is three months.

   [2]To prepare Rifampicin (Sigma, catalogue number R3501) stock solution (1%) dissolve 1 g of Rifampicin in 100 ml DMSO under stirring and store at room temperature protected from light. The shelf live is three months. To prepare Nalidixic acid and Rifampicin-containing agar plates (5µg of Nalidixic acid and 100 µg of Rifampicin/ml) add 0.25ml Nalidixic acid **and** 5ml of Rifampicin stock solution (1%) to 500 ml of dissolved hand-warm (50°C) BPLS agar. Store at temperature at 2°-8°C for not longer than 2 weeks.

   [3]To prepare Streptomycin (Sigma, catalogue number S9137) stock solution (1%) dissolve 1 g of Streptomycin in 100ml aqua dest. and store at 2°-8°C. The shelf live is three months. To prepare Streptomycin containing plates (200µg of Streptomycin/ml) add 10ml of Streptomycin stock solution (1%) to 500ml dissolved hand-warm (50°C) BPLS agar. Store at temperature at 2°-8°C for not longer than 2 weeks.

4. **Evaluation**

   Growth of *Salmonella*-like colonies are confirmed by means of BIOCHEMICAL DETERMINATION OF GENUS (to tell whether is *Salmonella* genus or not), followed by SEROTYPING METHOD (characterization based on sero-agglutination between vaccine flagella antigen and the commercial antisera for groups B & D). To be very sure, the sensitivity against Erythromycin of both vaccine strains can be verified.

For further information, please contact your Elanco representative. Not all products may be registered and available in all regions. Before using Elanco vaccines please consult your national compendium or seek advice from your Elanco representative or your veterinarian for the correct licensed use in your country. Elanco, AviPro™ and the diagonal bar are all trademarks owned or licensed by ELANCO, its subsidiaries or affiliates.
