# Supplementary material for: Effect of Antibiotics on the Colonization of Live Attenuated Salmonella Enteritidis Vaccine in Chickens
Source: Front Vet Sci. 2021 Dec 1;8:784160. doi: 10.3389/fvets.2021.784160 (PMC8671454; doi:10.3389/fvets.2021.784160)
Supplement: Supplementary file 2 [file Data_Sheet_2.pdf]

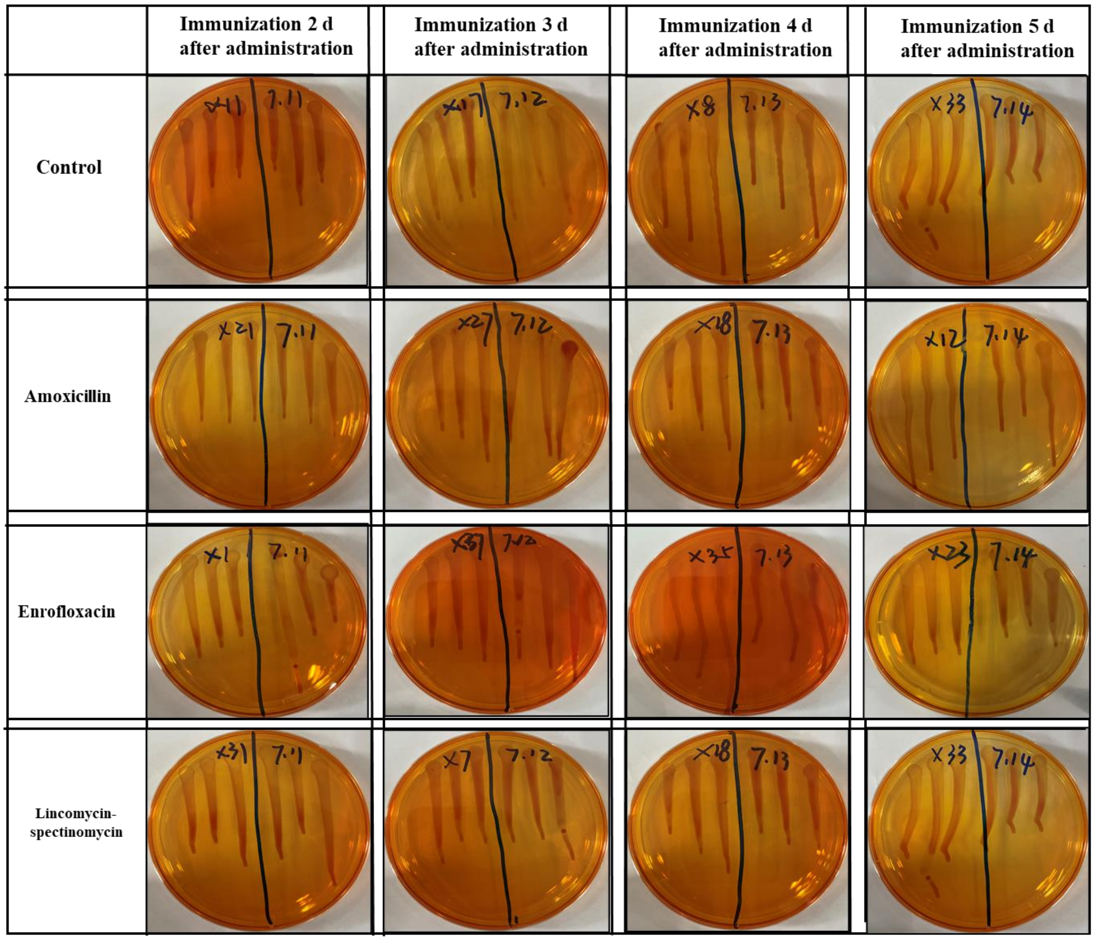

Figure S1. Results of Salmonella isolated from blood on BPLS medium in the amoxicillin, enrofloxacin and lincomycin-spectinomycin groups.

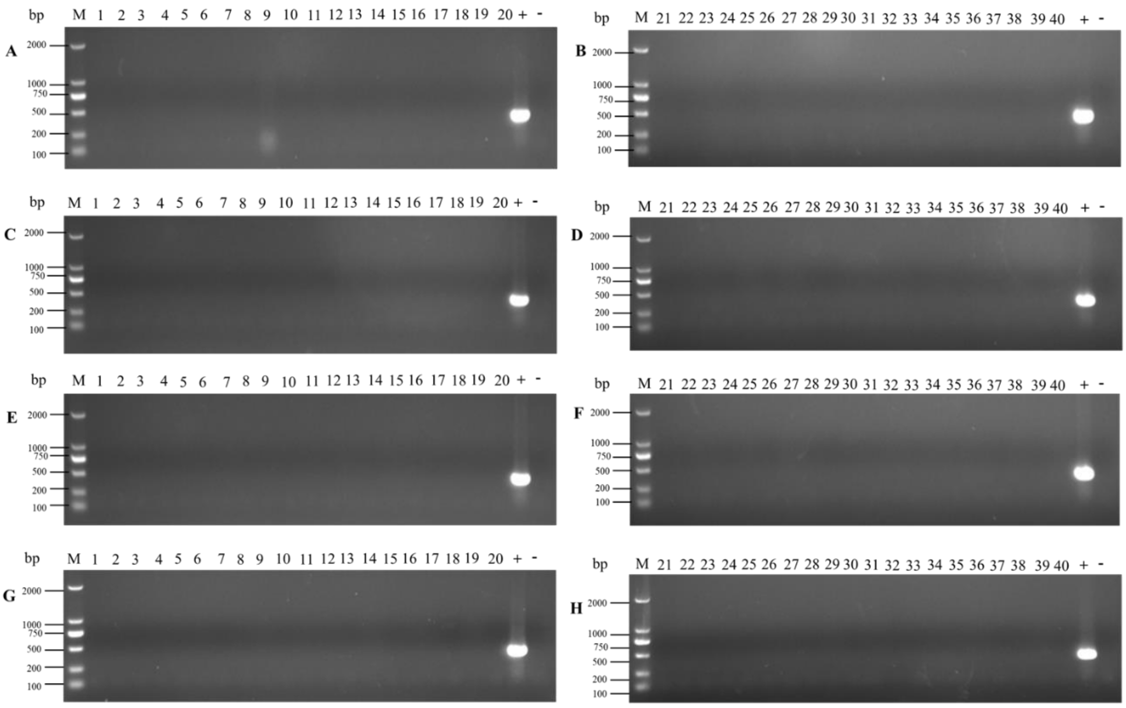

Figure S2. PCR detection of *Salmonella* in the blood in the amoxicillin, enrofloxacin and lincomycin-spectinomycin groups.

(A) Immunization 2 d after withdrawal, 1-10 control group, 11-20 amoxicillin group, + positive control, - negative control. (B) Immunization 2 d after withdrawal, 21-30 enrofloxacin group, 31-40 lincomycin-spectinomycin group, + positive control, - negative control. (C) Immunization 3 d after withdrawal, 1-10 control group, 11-20 amoxicillin group, + positive control, - negative control. (D) Immunization 3 d after withdrawal, 21-30 enrofloxacin group, 31-40 lincomycin-spectinomycin group, + positive control, - negative control. (E) Immunization 4 d after withdrawal, 1-10 control group, 11-20 amoxicillin group, + positive control, - negative control. (F) Immunization 4 d after withdrawal, 21-30 enrofloxacin group, 31-40 lincomycin-spectinomycin group, + positive control, - negative control. (G) Immunization 5 d after withdrawal, 1-10

control group, 11-20 amoxicillin group, + positive control, - negative control. (H) Immunization 5 d after withdrawal, 21-30 enrofloxacin group, 31-40 lincomycin-spectinomycin group, + positive control, - negative control.

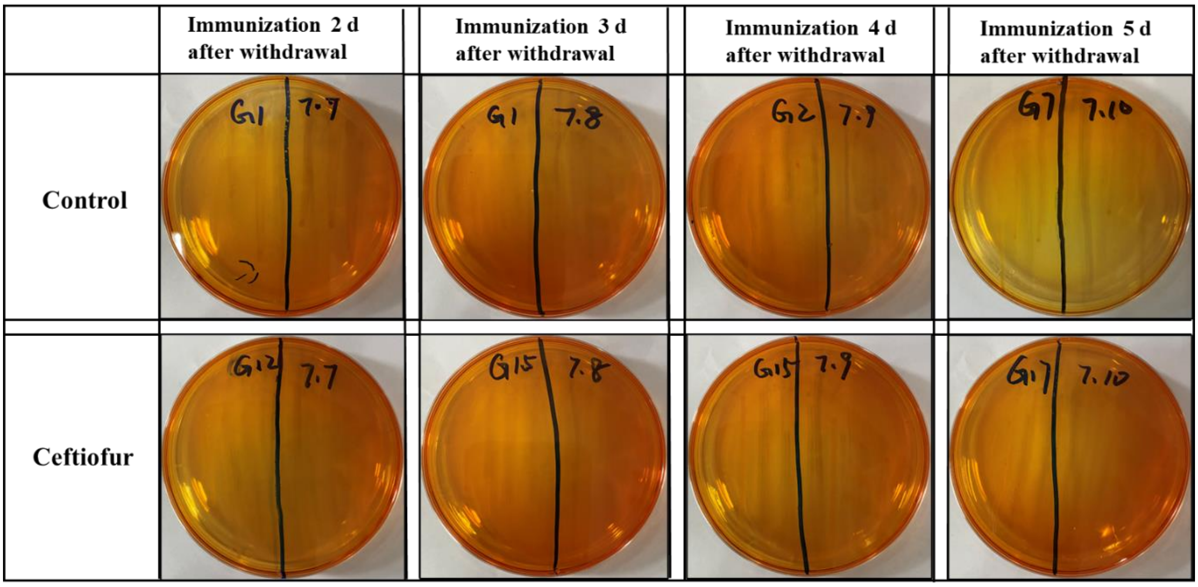

Figure S3. Results of *Salmonella* isolated from liver on BPLS medium in ceftiofur group.

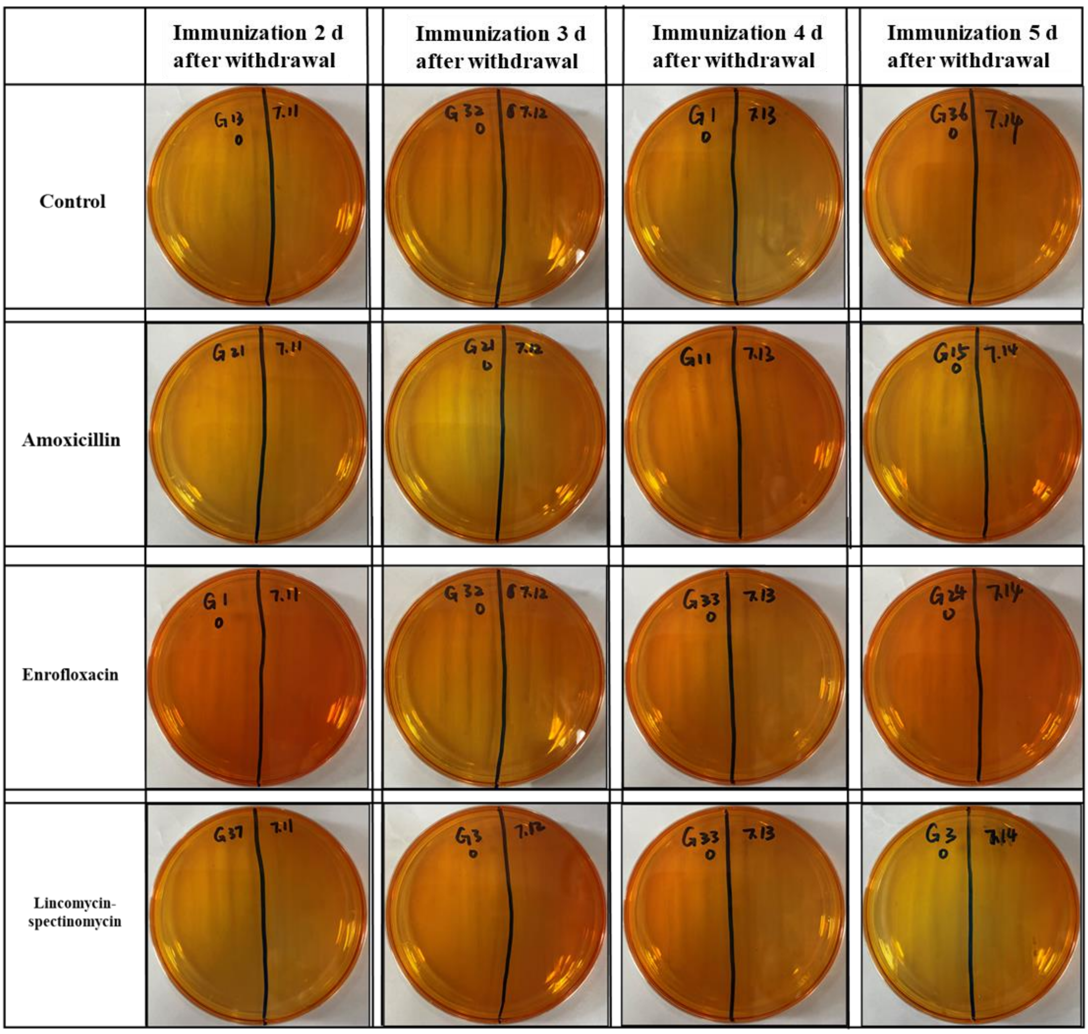

Figure S4. Results of *Salmonella* isolated from liver on BPLS medium in the amoxicillin, enrofloxacin and lincomycin-spectinomycin groups.

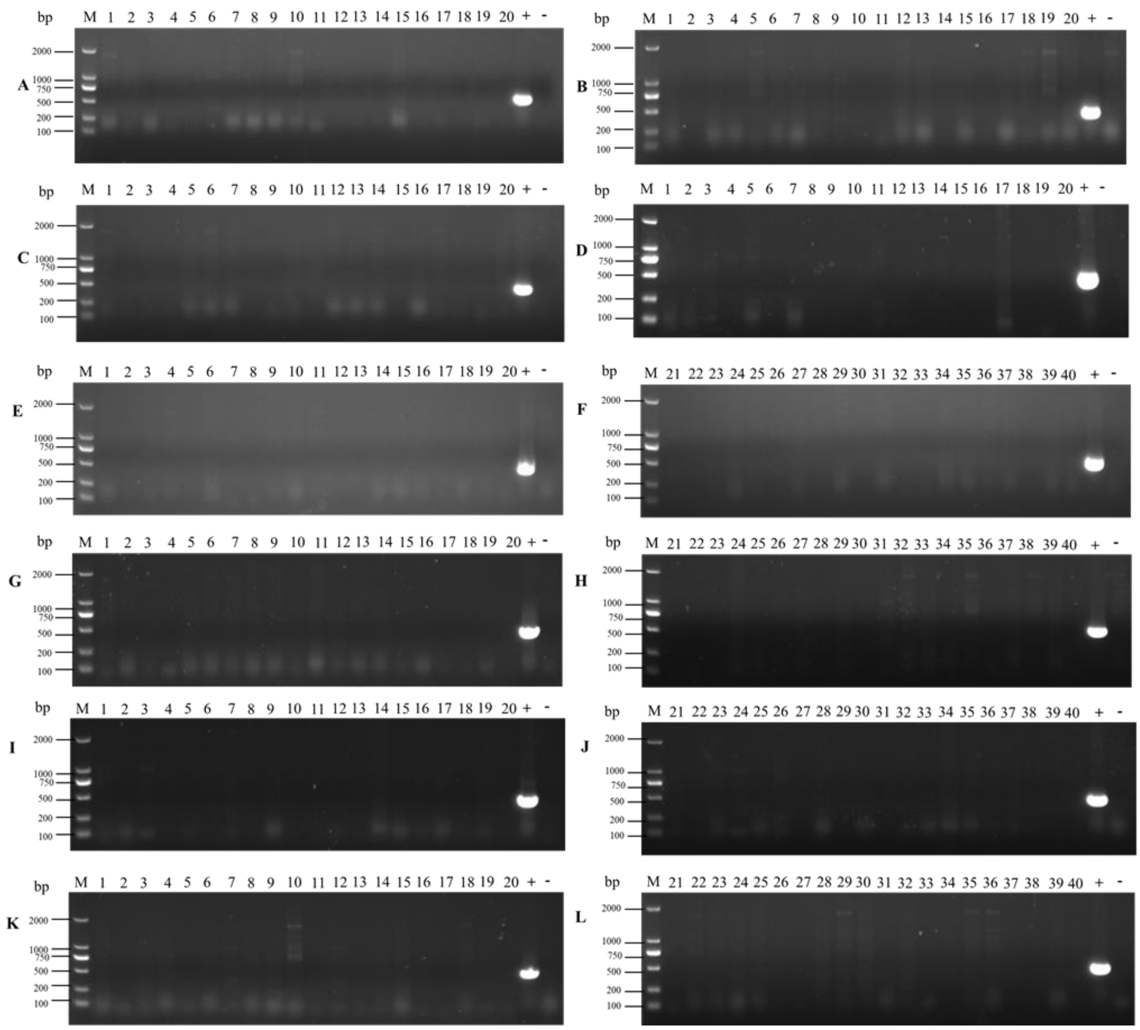

Figure S5. PCR detection of *Salmonella* in the blood in the ceftiofur, amoxicillin, enrofloxacin and lincomycin-spectinomycin groups.

(A) Immunization 2 d after withdrawal, 1-10 control group, 11-20 ceftiofur group, + positive control, - negative control. (B) Immunization 3 d after withdrawal, 1-10 control group, 11-20 ceftiofur group, + positive control, - negative control. (C) Immunization 4 d after withdrawal, 1-10 control group, 11-20 ceftiofur group, + positive control, - negative control. (D) Immunization 5 d after withdrawal, 1-10 control group, 11-20 ceftiofur group, + positive control, - negative control. (E) Immunization 2 d after withdrawal, 1-10 control group, 11-20 amoxicillin group, + positive control, - negative control. (F) Immunization 2 d after withdrawal, 21-30 enrofloxacin group, 31-40

lincomycin-spectinomycin group, + positive control, - negative control. (G) Immunization 3 d after withdrawal, 1-10 control group, 11-20 amoxicillin group, + positive control, - negative control. (H) Immunization 3 d after withdrawal, 21-30 enrofloxacin group, 31-40 lincomycin-spectinomycin group, + positive control, - negative control. (I) Immunization 4 d after withdrawal, 1-10 control group, 11-20 amoxicillin group, + positive control, - negative control. (J) Immunization 4 d after withdrawal, 21-30 enrofloxacin group, 31-40 lincomycin-spectinomycin group, + positive control, - negative control. (K) Immunization 5 d after withdrawal, 1-10 control group, 11-20 amoxicillin group, + positive control, - negative control. (L) Immunization 5 d after withdrawal, 21-30 enrofloxacin group, 31-40 lincomycin-spectinomycin group, + positive control, - negative control.
